# Aridity and land use negatively influence a dominant species' upper critical thermal limits

**DOI:** 10.7717/peerj.6252

**Published:** 2019-01-10

**Authors:** Nigel R. Andrew, Cara Miller, Graham Hall, Zac Hemmings, Ian Oliver

**Affiliations:** 1 School of Environmental and Rural Science, University of New England, Armidale, NSW, Australia; 2 School of Science and Technology, University of New England, Armidale, NSW, Australia; 3 Office of Environment and Heritage, Armidale, NSW, Australia

**Keywords:** Formicidae, Climate change, Landscape adaptation, Land cover, Land use, Thermal stress, Critical thermal maximum, Critical thermal minimum, Insect, Ant

## Abstract

Understanding the physiological tolerances of ectotherms, such as thermal limits, is important in predicting biotic responses to climate change. However, it is even more important to examine these impacts alongside those from other landscape changes: such as the reduction of native vegetation cover, landscape fragmentation and changes in land use intensity (LUI). Here, we integrate the observed thermal limits of the dominant and ubiquitous meat ant *Iridomyrmex purpureus* across climate (aridity), land cover and land use gradients spanning 270 km in length and 840 m in altitude across northern New South Wales, Australia. Meat ants were chosen for study as they are ecosystem engineers and changes in their populations may result in a cascade of changes in the populations of other species. When we assessed critical thermal maximum temperatures (CT_max_) of meat ants in relation to the environmental gradients we found little influence of climate (aridity) but that CT_max_ decreased as LUI increased. We found no overall correlation between CT_max_ and CT_min_. We did however find that tolerance to warming was lower for ants sampled from more arid locations. Our findings suggest that as LUI and aridification increase, the physiological resilience of *I. purpureus* will decline. A reduction in physiological resilience may lead to a reduction in the ecosystem service provision that these populations provide throughout their distribution.

## Introduction

As temperature and rainfall patterns are becoming less predictable and more variable across many parts of the world (Coumou & Rahmstorf, 2012; Harris et al., 2018), understanding population and species responses are critical to understanding how ecosystem structure will change (Smith, 2011a, 2011b). Understanding the impacts on species from exposure to extreme temperatures is critical, particularly for ectotherms and poikilotherms. It is important because these taxa, particularly insects, are more susceptible to seasonal variation and extreme temperature exposure, rather than the average annual increases in temperature (Clusella-Trullas, Blackburn & Chown, 2011; Kingsolver, Diamond & Buckley, 2013).

Evapotranspiration is a key defining measure of ecosystems (Stephenson, 1998). Changes in precipitation patterns occur with rising temperatures, increasing rates of evapotranspiration (Dai, 2011) which can lead to aridification (Girvetz & Zganjar, 2014). Many organisms can survive (Chanthy et al., 2015) or even thrive with high (or low) temperatures if there is an appropriate level of available moisture (Punzo & Mutchmor, 1980; Williams, Henry & Sinclair, 2014). However, in ecosystems with high temperatures and low available moisture (e.g. more arid environments), most individuals are less likely to perform well (Punzo, 1991) unless they have adapted to environmental water scarcity (Huang et al., 2015). Much of the research done along gradients, particularly latitudinal gradients, takes into account the direct measurement of temperature, but fewer take into account the relationship with moisture. Indeed at similar latitudes across continents precipitation and aridity plays a key role in species interactions and diversity pattern (Andersen, 2016; Byrne et al., 2008; Fattorini & Salvati, 2014; Pérez-Sánchez, Lattke & Viloria, 2013; Scherer et al., 2016; Suhling, Martens & Marais, 2009; VanDerWal et al., 2013; Wiens, Kozak & Silva, 2013; Yin, Ma & Wu, 2018). In this study, we use the composite Aridity index which incorporates data on both rainfall and evaporation.

It is intuitive to take into account the impacts of climate change relative to other anthropogenic changes to landscapes including the reduction of native vegetation cover, landscape fragmentation and changes in land use intensity (LUI; Oliver & Morecroft, 2014; Sala et al., 2000). Land-use change has led to major changes in biological patterns and process worldwide (Sala et al., 2000). In Australia, much of the landscape has changed, particularly since European settlement: native vegetation has been removed or highly modified for cropping or livestock grazing (Dorrough, Stoi & McIntyre, 2008). In some cases, the native vegetation has returned as secondary re-growth (Doherty, 1998). Land-use changes and changes in LUI can have immediate, short-term and long-term impacts on the soils and vegetation found at sites, and this can then modify the insect populations and communities that rely on these areas for shelter and food (Picanço et al., 2017; Tuck et al., 2014; Yates & Andrew, 2011; Yates, Gibb & Andrew, 2011).

To enable decision makers to make better decisions in regards to the management and conservation of biodiversity and ecosystem services both now and into the future, assessing the synergistic effects of changes in land cover, land use and climate are critical (Forister et al., 2010; Mawdsley, O’Malley & Ojima, 2009). Climate change is occurring in association with fragmenting landscapes and rapid movement of animals and plants locally, nationally and internationally, which all influence the evolutionary adaptation potential of species (Hoffmann & Sgro, 2011). Synergies among climate change and other key threats such as land clearing, land-use change and fragmentation (Brook, Sodhi & Bradshaw, 2008; Huey et al., 2012) have negative impacts on trees (Laurance & Williamson, 2001), ants via urbanisation (Diamond et al., 2017) and modelled species (Travis, 2003). Oliver et al. (2016) found that a greater amount of woody plant canopy cover increased ant richness (species and genus) and diversity, whereas a higher amount of land cultivation, grazing, exotic plant groundcover and bare ground reduced species richness. At sites with warmer and drier climates (i.e. a higher aridity index), native plant canopy cover had the greatest benefit, and exotic plant cover had the most negative effects, on ant species richness (Oliver et al., 2016). From the previous findings of Oliver et al. (2016), we predict that the effects of landscape change on diversity may also affect the thermal physiology of insect populations.

If local populations do not have large dispersal distances, they must adapt to their local conditions. In a warming climate, ectotherms may change their behaviours to find shade for heat relief or foraging away from exposed sites, forage at different times of the day (Kearney, 2013), or maintain a univoltine life-cycle to reduce exposure particularly in highly seasonal environments (Kearney et al., 2018). Thus, thermal physiology is a major constraint on how organisms cope with environmental change (Pörtner & Farrell, 2008).

Temperature mediates the physiological reactions of poikilotherms (Angilletta, 2009). Assessing their performance and physiological responses are critical to understanding biotic responses to climate change (Andrew & Terblanche, 2013), particularly the effects of exposure to thermal stress and temperature extremes (Andrew et al., 2013a; Vasseur et al., 2014). Biochemical and physiological reactions that are mediated by temperature and thermal stress can negatively influence development, growth, metabolism, movement and reproduction, leading to changes in community and ecosystem level processes (Dell, Pawar & Savage, 2011; Grigaltchik, Ward & Seebacher, 2012). Thermal performance curves identify how body temperature influences performance or fitness of an ectotherm (Sinclair et al., 2016). A key response to thermal stress identified by these curves is critical thermal limits: the functional endpoint that identifies upper and lower limits of temperatures that insects can tolerate from which they are unable to escape (Lighton & Turner, 2004). Assessing critical endpoints, such as critical thermal maximum (CT_max_) and critical thermal minimums (CT_min_), play a key role in understanding how insects can live and survive in their environments, but their calculations can vary widely (Terblanche et al., 2007). New statistical methods have been devised to attempt to generate consistency across methods (Kingsolver & Umbanhowar, 2018), especially as they now are being used for large-scale assessments (Sunday et al., 2014). Thermal stress is a key issue for all taxa including those that are dominant within ecosystems (Andrew, 2013; Andrew, Ghaedi & Groenewald, 2016; Andrew et al., 2013a; Mooney et al., 2009). Critical thermal limits of dominant and widespread species may change across aridity, climatic, land use and land cover gradients (Angilletta et al., 2007): leading to changes in community structure and the provision of ecological services (Traill et al., 2010; Stuble et al., 2014). Exposure to different microclimates may influence ectotherm physiology in more unpredictable ways than just exposure to warmer temperatures. Microclimates that ants are exposed to (Andrew et al., 2013a; Hemmings & Andrew, 2017) change substantially across surfaces within different habitat spaces: such as those with the bare ground, a high grazing intensity, exotic plant species cover and woody ground cover.

Throughout many terrestrial ecosystems worldwide, ants provide key ecosystem services and mediate key ecosystem processes (Del Toro, Ribbons & Pelini, 2012; Hölldobler & Wilson, 1990). Ants play key roles as ecosystem engineers in their environment and can influence the soil processes and properties surrounding their nests (Frouz & Jílková, 2008). Here, we focus on meat ants (*Iridomyrmex purpureus* (Smith, 1858) as they are a dominant and ubiquitous part of the landscape (Andersen, 2000; Greaves, 1971; Greenslade, 1976). *I. purpureus* populations are found throughout most of mainland Australia (ALA, 2018) and are a common and easily recognisable invertebrate (Greaves, 1971), primarily due to the large mounds they produce on the ground surface in open woodlands (Greaves & Hughes, 1974) and arid environments (Mobbs et al., 1978). They are behaviourally dominant as an actively aggressive diurnal species (Andersen & Patel, 1994).

*Iridomyrmex purpureus* can have a substantive impact on the availability of resources and the use of these resources by other species in different landscapes (Gibb, 2005). They are also excellent at resource exploitation and interference competition to enable them to dominate and control resources quickly (Gibb & Hochuli, 2004). Individual workers can maximise their foraging times by displaying opportunistic thermal responses and adjusting foraging behaviour to deal with high trail temperatures (Andrew et al., 2013a). Interestingly, throughout Australia, soil type is thought to limit their distribution, even if the climate envelope of the location is suitable (Greaves, 1971).

Our study was carried out along a 270 km aridity gradient spanning 840 m in altitude in northern NSW, Australia (Table 1). We predict that a range of environmental stressors will influence meat ants CT_max_, but aridity will be the key driver, and that CT_min_ should be relatively consistent along the gradient. Warming tolerance defines how much warming an organism can tolerate before lethal levels are attained (Deutsch et al., 2008): it measures the difference between the upper critical thermal limit and the ambient habitat temperature. These values can change substantially based on the derivation method of habitat temperatures (Andrew et al., 2013a). Warming tolerance of meat ants should be highest at the cooler/wetter parts of the aridity gradient, and the calculated measure should consistently reduce if warming tolerance is based on either (1) data from long-term climatic data, (2) seasonal data or (3) daytime temperature data when the ants are foraging.

**Table 1:** Characteristics of the 11 sites used in this study (from a total of 87).

Site name	Aridity	Altitude	Land use intensity	Soil clay	Total native woody cover	Exotic ground cover	pH	Lat	Long
Smokey Mountain (38)	0.401	891	2	5	23	50	4.585	−29.966	151.271
Furrocabad Station (44)	0.346	1,047	3	28.8	30	69	5.25	−29.83	151.608
Furrocabad Station (45)	0.366	1,008	8	28.8	97	68	5.64	−29.823	151.598
Delunga 52	0.617	338	0	37.5	76	9	6.16	−29.835	150.554
62	0.716	203	4	27.5	16	43	5.675	−29.379	149.797
63	0.715	204	5	30	66	2	5.855	−29.378	149.796
87C	0.732	163	6	57.5	1	83	6.425	−29.693	149.23
Towarra (96)	0.537	643	1	28.8	70	1	5.56	−30.125	150.76
Myall Creek (117)	0.583	457	1	65	68	18	6.465	−29.823	150.74
West Oaks (126C)	0.491	730	4	15	97	2	5.89	−29.359	151.429
West Oaks (127C)	0.508	683	5	53.8	1	10	5.695	−29.36	151.412

**Note:**

See Oliver et al. (2016) for details on the full complement of sites.

Here, we address the following questions:

What are the critical thermal limits (CT_max_ and CT_min_) for *I. purpureus* across sites representing the main climatic, vegetation and land-use regimes?
What are the key environmental drivers (climatic, soil, vegetation and land-use regimes) influencing thermal limits?
What is the relationship between *I. purpureus’* warming tolerance and aridity along the environmental gradient?

## Methods

### Site selection

A total of 11 sites were chosen from the 87 used in Oliver et al. (2016) to sample the range of climatic, land-use and native woody vegetation cover along these gradients (Table 1). The area has some of the most fertile soils in Australia, with much of the farming practices dominated by livestock grazing on modified pastures and native vegetation, and dryland and irrigated cropping (Australian Bureau of Rural Sciences (BRS), 2009). Semi-arid woodlands dominate native remnant vegetation at lower altitudes through to grassy woodlands and dry sclerophyll forest at higher altitudes (Keith, 2004). Sites were chosen to maximise the range in: climate (Aridity: based on rainfall and evaporation collected from modelled climate data from ANUCLIM 6.1 (Xu & Hutchinson, 2011) over three time periods: 3 months, 12 months and 36 months); land cover (total native woody cover (Canopy) and bare ground); land use (intensity of use: LUI; exotic groundcover); soil pH; and clay content. LUI is a semi-quantitative index based on cultivation and grazing severity and age: so more intensively managed sites have higher values (ranging between 0 and 12). More information on the calculation and justification of using these variables can be found in Oliver et al. (2016). Aridity was calculated as:

$${\rm{Aridity}} = 1-{{{\rm{Rain}}} \over {{\rm{Evap}}}}$$

Where *Rain* and *Evap* are the total rainfall and pan evaporation in millimetre for the period of interest. Aridity ranges from 0 to 1 with more arid environments approaching 1. The aridity index had a high correlation with temperature over three time periods (3 months, 12 months and 36 months) generated using from ANUCLIM 6.1 (Correlation R above 97.4% and *p* > 0.0001 for all pairwise comparisons) among sites. Therefore, we chose to keep the direct model comparisons with aridity.

Collections of a minimum of 30 individual *I. purpureus* from each site occurred between April and May 2014. After collection, ants were held at 25 °C for 2 h to avoid effects of time of day of capture differences along the gradient. Previous work on *I. purpureus* found no effect of time of day of capture/nest temperature on thermal tolerances (determined via thermolimit respirometry) from a single site (Andrew, Ghaedi & Groenewald, 2016).

### Critical thermal maximum and minimum assessments (CT_max_ and CT_min_)

CT_max_ measurements were carried out in a Grant R4 waterbath with a GP200 heater using distilled water. A total of 10 individuals from each site were each put into a single 50 ml vial for testing, and readings for each ant were taken. The waterbath temperature was stabilised for 10 min at 25 °C and then ramped at 0.25 °C/min until CT_max_ was reached. CT_max_ was identified when an individual ant could not perform coordinated motor functions in the vial to right itself after being turned onto its side (Andrew et al., 2013a). CT_max_ could go up to 55 °C (equivalent to 120 min/individual and 30 °C temperature change at 0.25 °C/min). Ramping at 0.25 °C is considered the most ‘standard’ temperature ramping rate, at which the body temperature of ants is in equilibrium with their surroundings (Andrew et al., 2013a; Chown et al., 2009; Lighton & Turner, 2004; Nguyen et al., 2014; Terblanche et al., 2007). CT_min_ was carried out similarly to CT_max_ using 1:1 distilled water/glycol mix. Waterbath temperature was stabilised for 10 min at 5 °C and then decreased at a rate of 0.25 °C/min until CT_min_ was reached. CT_min_ was identified when an individual ant could not perform coordinated motor functions in a 50 ml vial to right itself after being turned onto its side (Andrew et al., 2013a). CT_min_ could go down to −15 °C (equivalent to 80 min/species and 20 °C temperature change at 0.25 °C/min). To measure temperatures that ants were exposed to within each vial, a Type-T thermocouple was placed within another 50 ml vial that was plunged with the ants and connected to a temperature datalogger (Testo 175 T3; Testo, Melbourne, Australia) with data logged as waterbath temperatures were ramped: the Testo temperature was used to identify ant CT_max/min_.

### Model fitting

We used *R* (R Core Team, 2017) and the R package *lme4* (Bates et al., 2015) to perform a linear mixed effects analysis of the relationship between CT_max_ as a response variable against the environmental variables of aridity, LUI (converted to a proportion), soil clay content, exotic plant ground cover and total native woody cover (Canopy) designated as fixed effects. We explored the singular interaction effects of Aridity:LUI, Canopy:LUI and Clay:LUI in some models as well as the impact of dropping main effect variables. With this framework, we considered random intercept models by site only, and by both site and CT_min_ (individually). We also considered a random intercept, random slope model with CT_min_ within Site as the random effect. We repeated this model selection process with CT_min_ as the response variable and CT_max_ as the predictor variable where appropriate. Models were initially fit with REML and then refitted with ML for comparison in Likelihood ratio tests. Minimum AIC values and *p*-values < 0.05 were used to aid model selection. Visual inspection of residual plots of the preferred models was used to assess obvious deviations from homoscedasticity or normality. Visualisation of random effects was undertaken using R package *sjPlot* (Lüdecke, 2017). Standard errors and confidence intervals for predicted values of preferred models were undertaken using parametric bootstrapping (*n* = 1,000) within R package *bootpredictlme4* (Duursma, 2017) and visualised within R package *visreg* (Breheny & Burchett, 2017).

### Warming tolerance

Warming tolerance was calculated using the equation of Deutsch et al. (2008) and Diamond et al. (2012): CT_max_ − T_hab_. The T_hab_ calculation may include different calculations (e.g. annual average; summer average; microclimate summer average; and microclimate summer 10 am–4 pm summer average) which are ecologically relevant and to identify the most appropriate to assess ectotherm stress (Andrew et al., 2013a). For T_hab_ here, we did not have access to microclimate data, so we modelled site location data using ANUCLIM V6.1 (Xu & Hutchinson, 2011) from the closest weather stations based on 3 months summer average 2009, 12-month average for 2009 and 36 months (2007–2009) average day temperatures. These weather data were used, as the data were generated for all sites at the time of sampling ant species richness in Oliver et al. (2016).

The data files used in this assessment can be found on Figshare (Andrew et al., 2018).

## Results

**Figure 1:**
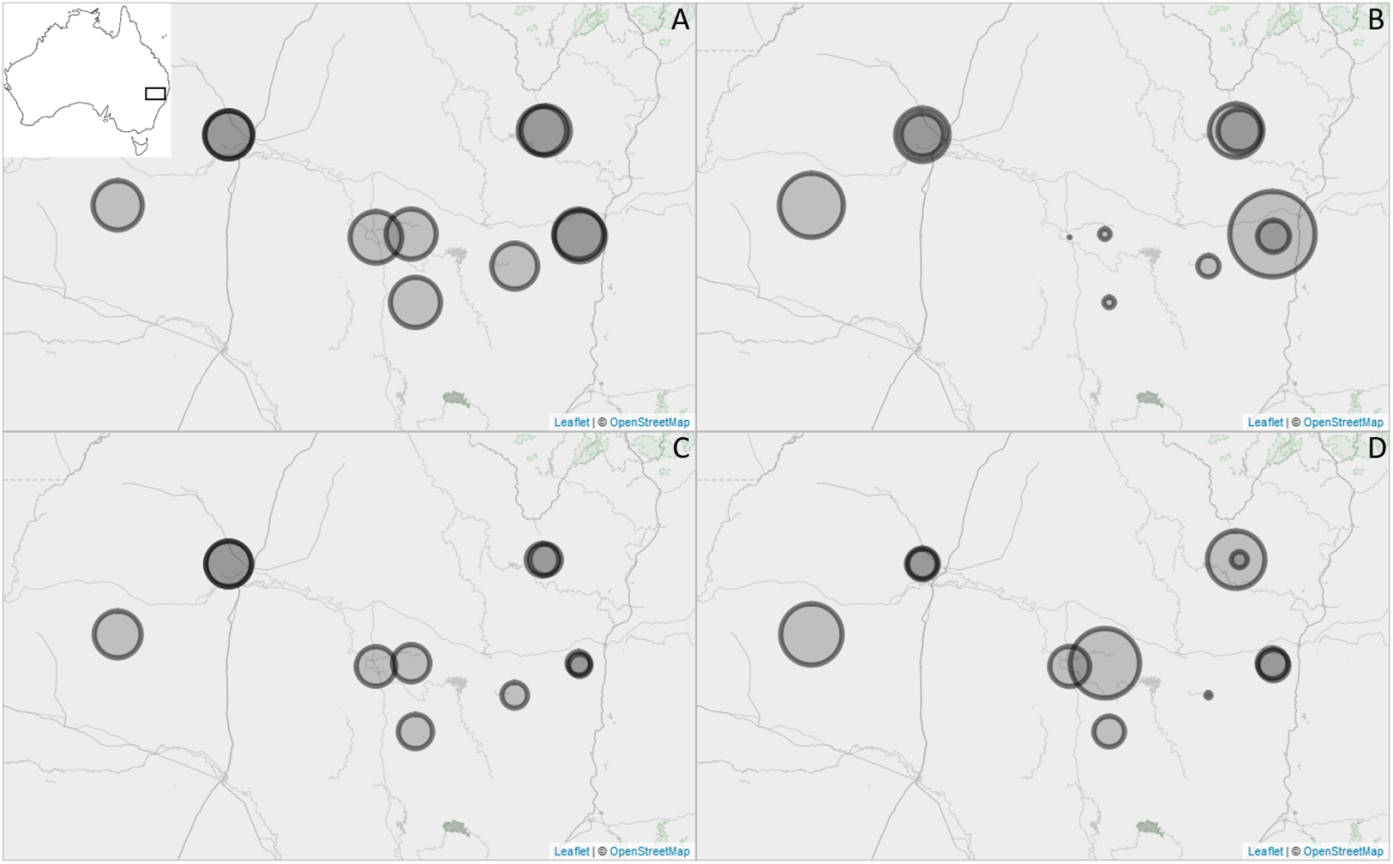
Sites used in this study in northern New South Wales, Australia (see insert) with relative values for CT_max_ (A), land use intensity (B), aridity (C) and clay (D) shown. Maps generated using Map data © OpenStreetMap contributors. The size of the circle is indicative of the mean value of the given variable (i.e. the larger the circle, the higher the value and the contrary). Image produced using the Leaflet package (version 1.1.0.9000, http://rstudio.github.io/leaflet/) within R statistical software (version 3.4.3). The R package OpenStreetMap is licensed under a GNU General Public License (GPL-2) (https://cran.r-project.org/web/packages/OpenStreetMap/index.html) and was used to extract map tiles from OpenStreetMap which is licensed on terms of the Open Database License, ‘ODbL’ 1.0. (http://wiki.osmfoundation.org/wiki/Licence).

Critical thermal maxima of individual ants ranged between 41.5 and 49.2 °C, and CT_min_ between 0.3 and 7.1 °C in this study. There was no consistent relationship between CT_min_ and CT_max_ across the 11 sites sampled (Fig. 1), suggesting no causal relationship between the two endpoints.

The preferred model proposed for explaining meat ants CT_max_ across the landscape is:

$${\rm{C}}{{\rm{T}}_{{\rm{max}}}}\sim{\rm{ LUI }} + {\rm{ Canopy }} + {\rm{ Exotic }} + {\rm{ Clay }} + {\rm{ Aridity }} + {\rm{ }}\left({{\rm{C}}{{\rm{T}}_{{\rm{min}}}}{\rm{|SITE}}\_{\rm{ID}}} \right)$$

**Figure 2:**
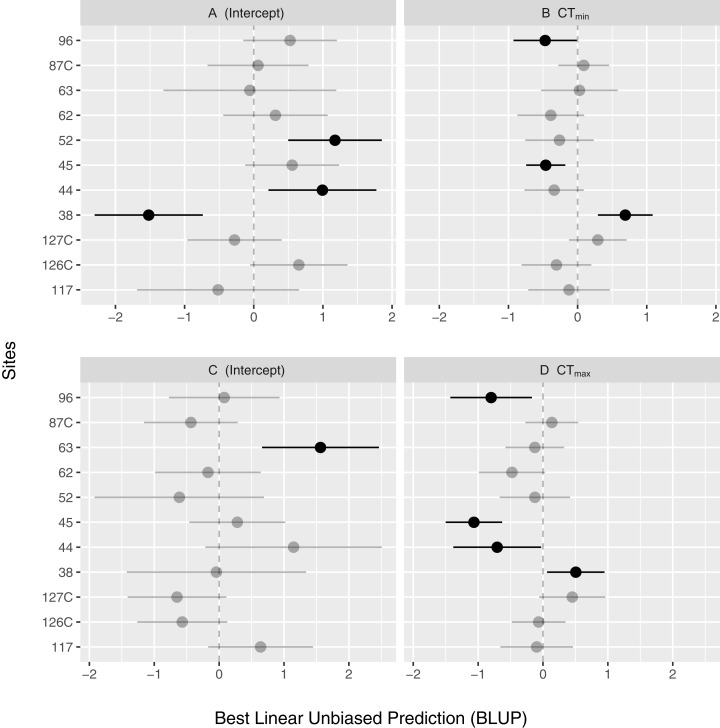
Random effect estimates of model coefficients using best linear unbiased prediction (BLUP) and 95% confidence intervals of the intercepts (A and C) and CT_max_ (B) and CT_min_ (D) across sites.

**Figure 3:**
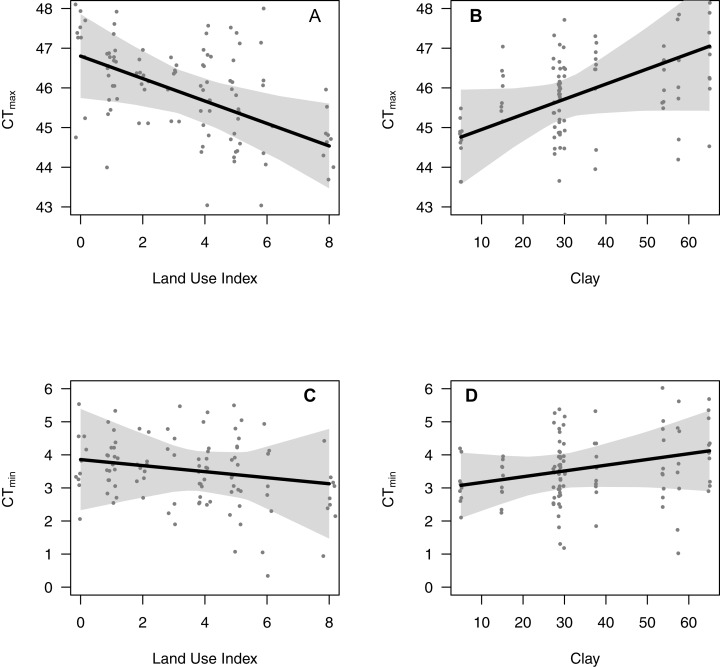
95% confidence intervals on the selected CT_max_ and CT_min_ models for the factors: land use intensity (LUI: A and C) and clay (B and D).

The fixed effects for this model are shown in Table 2. The overall random effects for the model above are (in terms of variance): Site: 1.04; CT_min_|Site: 0.19; and Residuals: 1.49. As shown in Fig. 2, variation among Sites is an important source of variation (much more so than CT_min_ although the inclusion of this was still significant as per the model selection process). However, there is still additional (unaccounted for) variation in the residuals. For the variables of LUI and Clay, there were significant relationships with CT_max_ (Fig. 3). As LUI increases, CT_max_ decreases; whereas clay content was positively correlated with CT_max_.

**Table 2:** Estimated fixed effects for the selected CT_max_ model.

	Estimate	Standard error	2.5%	97.5%
(Intercept)	46.04	0.30	45.52	46.54
Land use intensity (LUI)	−0.28	0.15	−0.48	−0.06
Total native woody cover (Canopy)	0.01	0.01	−0.014	0.02
Exotic groundcover	0.01	0.01	−0.01	0.03
Soil clay content	0.04	0.02	0.01	0.06
Aridity index	2.82	2.54	−6.37	0.18

**Note:**

Standard errors and 95% confidence intervals are also presented. All variables have been centred.

For explaining meat ants CT_min_ across the landscape, a similar model is proposed as that for CT_max_:

$${\rm{CT}}_{{\rm{min}}} \sim {\rm{ LUI }} + {\rm{ Canopy }} + {\rm{ Exotic }} + {\rm{ Clay }} + {\rm{ Aridity }} + {\rm{ }}\left({{\rm{C}}{{\rm{T}}_{{\rm{max}}}}{\rm{|SITE}}\_{\rm{ID}}} \right)$$

The fixed effects for this model are shown in Table 3. The overall random effects for the model above are (in terms of variance): Site: 1.3; CT_max_|Site: 0.35; Residuals: 1.38. As with CT_max_, the sites also exhibit a high amount of variation (Fig. 2). The prediction intervals for CT_min_ also show similar results as those for CT_max_ (Figs. 3C and 3D), however, the relationships are weaker for both LUI and Clay content.

**Table 3:** Estimated fixed effects for the selected CT_min_ model.

	Estimate	Standard error	2.5%	97.5%
(Intercept)	3.7	0.37	2.85	4.28
Land use intensity	−0.091	0.18	−0.34	0.15
Total native woody cover (Canopy)	0.00	0.013	−0.03	0.02
Exotic groundcover	0.004	0.016	−0.02	0.023
Soil clay content	0.017	0.025	−0.01	0.06
Aridity index	0.29	3.3	−4.24	5.01

**Note:**

Standard errors and 95% confidence intervals are also presented. All variables have been centred.

**Figure 4:**
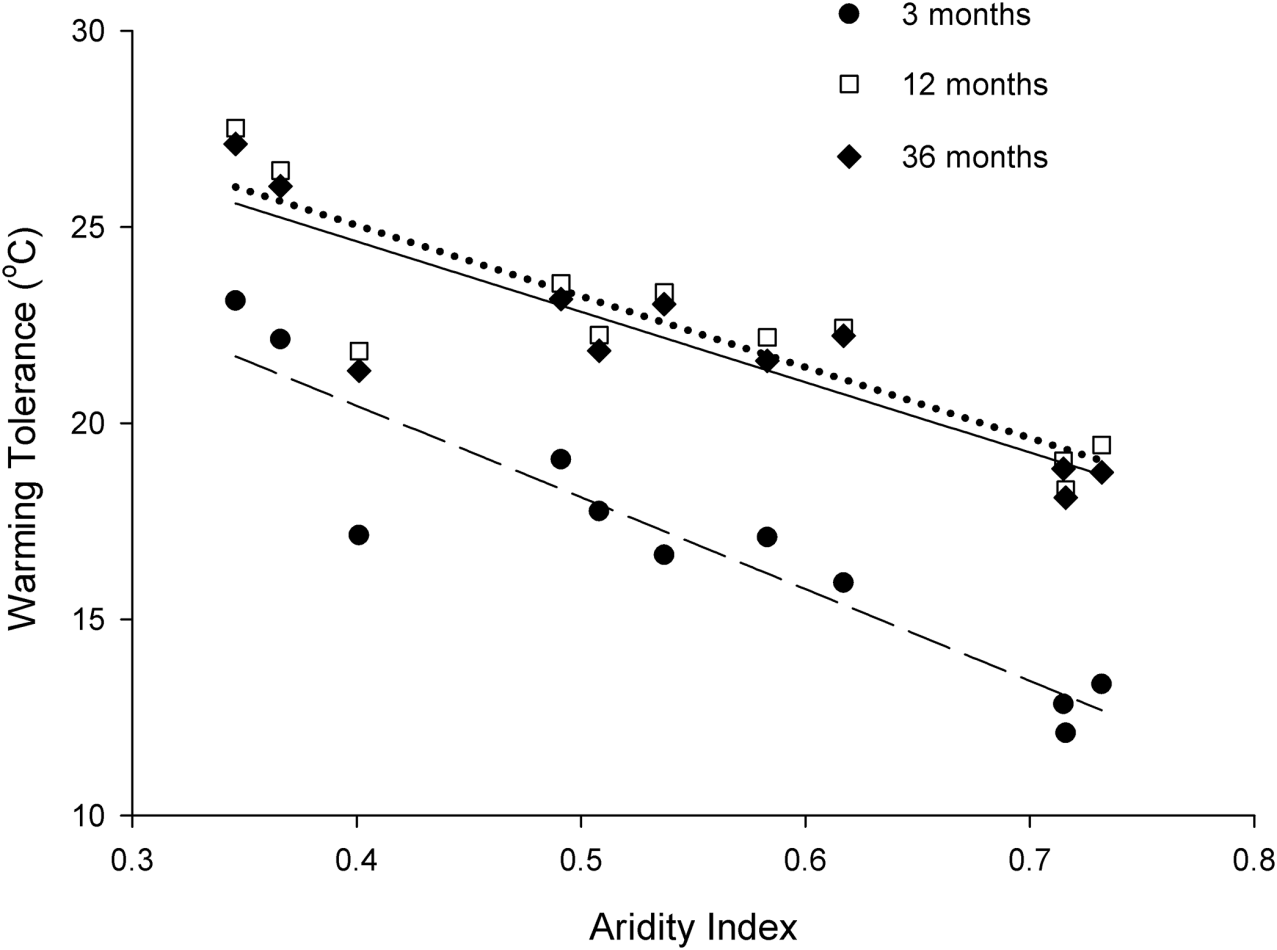
Three measures of warming tolerance (3 months, 12 months and 36 months); based on location modelled ANUCLIM data for three different sampling periods) relative to aridity index for each site.

We found a negative relationship between the warming tolerances of *I. purpureus* and landscape aridity (Fig. 4). This relationship was consistent among all measures of mean temperatures (no significant difference in Test for Common Slope across Groups: Test Statistic = 1.49, *p* = 0.482). There was a significant difference in the slope elevation of warming tolerance between the 3 months and 36 months mean temperature calculations (Fig. 4; d.*f*. = 2, WALD = 95.30, *p* < 0.0001).

## Discussion

Identification of critical thermal limits and physiological responses of insects to a changing climate is crucial for understanding how individuals and populations will respond to changes in their local environment (Andrew et al., 2013b; Andrew & Terblanche, 2013). These responses are becoming a key area of research interest (Andrew et al., 2013a). The assessment of common species responses to a changing climate needs to be thoroughly assessed, as changes in the population structure of these taxa can have large implications for the ecosystems in which they provide ecosystem services (Andrew, 2013; Gaston, 2011; Inger et al., 2014). Also, as landscapes become more fragmented and disturbed, common and dominant species responses to changes may also be limited. Here, critical thermal maxima and minima were determined for populations of a dominant ant species that encompassed an extensive distribution along an environmental gradient.

There was no strong pattern in CT_max_ and CT_min_ associated with the environmental variables tested. The results of the CT_max_ measurements indicate there is a high variation of CT_max_ across sites, this may be due to the ants being field fresh, and so their previous exposure to a variety of stresses may influence their thermal capabilities. However, this is also important, as it indicates that no one individual stress dominates the thermal abilities of *I. purpureus* workers on site. Meat ants are also highly adaptable: they are known to run faster back to the nest when exposed to surface temperatures above its critical thermal maximum (Andrew et al., 2013a); they are exposed to different microclimate temperatures throughout their microhabitats (Hemmings & Andrew, 2017); and they have different thermolimit responses based on exposure to microclimate temperatures on the nest surface (Andrew, Ghaedi & Groenewald, 2016).

Critical thermal maxima of individual ants ranged between 41.5 and 49.2 °C, and CT_min_ between 0.3 and 7.1 °C in this study. This is a very wide range of readings for CT_max_ and could be due to age, nutritional status, stress or prior heat exposure that the ants may have been exposed to (Nyamukondiwa & Terblanche, 2009; Sørensen, Dahlgaard & Loeschcke, 2001). Upper thermal limits are thought to be less variable compared to lower limits (Addo-Bediako, Chown & Gaston, 2000; Diamond et al., 2017), however, it is known that environmental exposure does influence these limits (Hoffmann, Chown & Clusella-Trullas, 2013), and this is seen in the relationships with both LUI and Clay in this study. Here, we measured CT_max_ and CT_min_ by observing an individual ant’s ability to right itself while temperatures were increasing at 0.25 °C/min. The calculation of critical thermal limits using ant righting behaviour may be more variable than using physiological critical limits. Physiological critical limits include methods such as upper lethal temperatures: where ants are exposed to static temperatures for 2 h (Andrew et al., 2013a); or thermolimit respirometry: where CT_max_ is derived from metabolic measurements using flow-through CO_2_-based respirometry and optical detection, when temperatures are ramped at a consistent rate (Andrew, Ghaedi & Groenewald, 2016; Lighton & Turner, 2004). As an alternative measure of CT_max_, thermolimit respirometry (Lighton & Turner, 2004) may be more robust, as the method explicitly measures the ceasing of metabolism (release of carbon dioxide) of the ant; but it is also a different measure of CT_max_, as there is no ability for the ants to recover from heat exposure in thermolimit respirometry.

When the fitted models were used to assess critical thermal limits, it was clear that site-specific differences strongly influenced the results found. However, LUI and soil clay content also played a significant role in influencing ant physiological end-points. This suggests that ant populations that were exposed to higher levels of habitat modification (via LUI) showed lower climatic resilience relative to less disturbed habitats. However, there is still additional unaccounted for variation in the residuals which suggests that there may be other variables (unmeasured) that may affect the meat ants’ climatic resilience.

For ants, much of the research on local effects of habitat disturbance has been carried out on changes in communities (Andersen & Majer, 2004; Andrew, Rodgerson & York, 2000; Bromham et al., 1999; Yates et al. 2014). Previous work carried out along the same gradient (Oliver et al., 2016) used for this study found clear evidence supporting a role for landscape adaptation to maintain and restore species richness of ant communities at the site level. Their study found that higher woody native tree and shrub cover, and lower exotic plant groundcover had a positive effect on ant species richness. Interestingly, LUI the authors found no significant impact on the species richness within any of the ant genera assessed across the gradient.

In this study, soil clay content had a positive effect on *I. purpureus* worker CT_max_. Soil clay content did have a positive influence on *Iridomyrmex* spp. diversity in Oliver et al. (2016). Clay and clay-like substrate is an important component for ant nest development (Monaenkova et al., 2015) and is critical for other insect taxa, such as termites in giving their feeding galleries structural integrity (Oberst, Lai & Evans, 2016). Soil type is known to influence meat ant distribution (Greaves, 1971), and clay soils have a much better ability to hold moisture (O’Geen, 2013). Meat ants are known to remove fine and medium grain sand from their nests (Ettershank, 1968) which has poorer moisture holding properties than clay (Saxton et al., 1986). As *I. purpureus* nests are known to remain in the same location for over 70 years (Greenslade, 1975), substantive structural elements are required to keep the nest maintained during this time. The amount of clay in an *I. purpureus* nest is representative of the surrounding non-nest soil (Ettershank, 1968). *I. purpureus* nests are also not found on quartz sand soils, even when climatic factors are suitable, indicating that soil type can be a limiting distributional factor (Greaves, 1971). As clay plays a role in the distribution of the species, it also plays a role in the physiological breadth of individuals.

We calculated warming tolerance using three different measures of habitat temperature; all generated based on location data using ANUCLIM. In line with our predictions these all indicated that an increase in aridity reduced ant tolerance to warming. When the warming tolerance was previously calculated for *I. purpureus* at a higher altitude (Armidale, NSW: 980 m.a.s.l.), similar calculations were made: a warming tolerance of 19.5 °C was calculated on weather station summer average, and 25.8 °C based on weather station annual average (Andrew et al., 2013a). As Armidale is a more temperate site than those tested here, it would be at the lower scale of the aridity index. Across the aridity index (which has a high correlation with temperature among sites), there was a 10 °C difference in warming tolerance for *I. purpureus*. With a prediction of global increases in air temperature of 2 and 6 °C over the 21st Century, and in the region assessed there is an 80% probability of a 3 °C warming and a 30% probability of a 4 °C warming with a likelihood of reduced annual rainfall of 3–5% (CSIRO-ABM, 2012), aridity of the region assessed will only continue to increase. With further temperature variation and exposure to extreme temperature events (Harris et al., 2018), especially at the microscale, these mid-latitude ants will become more susceptible to heat stress (Kingsolver, Diamond & Buckley, 2013).

## Conclusion

From this study, we found that across the range of our gradients, habitat type (e.g. soils) and LUI were more limiting factors on meat ant CT_max_ and CT_min_ than climate, but that an increase in aridity did reduce ant tolerance to warming. Meat ants are dominant and abundant within the ecosystems where they are found, and physiological stress may reduce their abundance and change their interactions with other species. As sharp declines in insect abundance are becoming increasingly well documented with changing climates (Lister & Garcia, 2018), and key changes in species richness with land-use change are documented (Oliver et al., 2016), it becomes increasingly important to better understand the relative and potentially synergistic influences of the biotic and abiotic environment (Andrewartha & Birch, 1954; Berggren et al., 2009; Chown & Terblanche, 2007) in causing these changes.

## Additional Information and Declarations

### Data Availability

The following information was supplied regarding data availability: Andrew, Nigel; Miller, Cara; Hall, Graham; Hemmings, Zac; Oliver, Ian (2018): Aridity and land use negatively influence a dominant species’ upper critical thermal limits. Dataset for PeerJ paper. figshare. Dataset. DOI 10.6084/m9.figshare.7367078.v1.

## References

[ref-1] Addo-Bediako A, Chown SL, Gaston KJ (2000). Thermal tolerance, climatic variability and latitude. Proceedings of the Royal Society B: Biological Sciences.

[ref-2] ALA (2018). Atlas of Living Australia *Iridomyrmex purpureus*. https://bie.ala.org.au/species/urn:lsid:biodiversity.org.au:afd.taxon:7d58c2f3-3ddd-4ebd-bab6-a58c9c367976.

[ref-3] Andersen AN (2000). The Ants of Northern Australia: a guide to the monsoonal fauna.

[ref-4] Andersen AN (2016). Ant megadiversity and its origins in arid Australia. Austral Entomology.

[ref-5] Andersen AN, Majer JD (2004). Ants show the way down under: invertebrates as bioindicators in land management. Frontiers in Ecology and the Environment.

[ref-6] Andersen AN, Patel AD (1994). Meat ants as dominant members of Australian ant communities: an experimental test of their influence on the foraging success and forager abundance of other species. Oecologia.

[ref-7] Andrew NR, Rohde K (2013). Population dynamics of insects: impacts of a changing climate. The Balance of Nature and Human Impact.

[ref-8] Andrew NR, Ghaedi B, Groenewald B (2016). The role of nest surface temperatures and the brain in influencing ant metabolic rates. Journal of Thermal Biology.

[ref-9] Andrew NR, Hart RA, Jung M-P, Hemmings Z, Terblanche JS (2013a). Can temperate insects take the heat? A case study of the physiological and behavioural responses in a common ant, *Iridomyrmex purpureus* (Formicidae), with potential climate change. Journal of Insect Physiology.

[ref-10] Andrew NR, Hill SJ, Binns M, Bahar MH, Ridley EV, Jung M-P, Fyfe C, Yates M, Khusro M (2013b). Assessing insect responses to climate change: what are we testing for? Where should we be heading?. PeerJ.

[ref-11] Andrew NR, Miller C, Hall G, Hemmings Z, Oliver I (2018). Aridity and land use negatively influence a dominant species’ upper critical thermal limits. Dataset for PeerJ paper. Figshare.

[ref-12] Andrew N, Rodgerson L, York A (2000). Frequent fuel-reduction burning: the role of logs and associated leaf litter in the conservation of ant biodiversity. Austral Ecology.

[ref-13] Andrew NR, Terblanche JS, Salinger J (2013). The response of insects to climate change. Living in a Warmer World: How a Changing Climate will Affect Our Lives.

[ref-14] Andrewartha HG, Birch LC (1954). The distribution and abundance of animals.

[ref-15] Angilletta MJ (2009). Thermal adaptation: a theoretical and empirical synthesis.

[ref-16] Angilletta MJ, Wilson RS, Niehaus AC, Sears MW, Navas CA, Ribeiro PL (2007). Urban physiology: city ants possess high heat tolerance. PLOS ONE.

[ref-22] Australian Bureau of Rural Sciences (BRS) (2009). Land use summary border rivers/Gwydir NRM Region—NSW. Catchment scale land use mapping for Australia. Update May 2009 dataset. Bureau of Rural Sciences. http://www.daff.gov.au/abares/aclump/pages/land-use/catchment-scale-land-use-reports.aspx.

[ref-17] Bates D, Mächler M, Bolker B, Walker S (2015). Fitting linear mixed-effects models using lme4. Journal of Statistical Software.

[ref-18] Berggren A, Bjorkman C, Bylund H, Ayres MP (2009). The distribution and abundance of animal populations in a climate of uncertainty. Oikos.

[ref-19] Breheny P, Burchett W (2017). https://CRAN.R-project.org/package=visreg.

[ref-20] Bromham L, Cardillo M, Bennett AF, Elgar MA (1999). Effects of stock grazing on the ground invertebrate fauna of woodland remnants. Australian Journal of Ecology.

[ref-21] Brook BW, Sodhi NS, Bradshaw CJA (2008). Synergies among extinction drivers under global change. Trends in Ecology & Evolution.

[ref-23] Byrne M, Yeates DK, Joseph L, Kearney M, Bowler J, Williams MAJ, Cooper S, Donnellan SC, Keogh JS, Leys R, Melville J, Murphy DJ, Porch N, Wyrwoll KH (2008). Birth of a biome: insights into the assembly and maintenance of the Australian arid zone biota. Molecular Ecology.

[ref-24] Chanthy P, Martin B, Gunning R, Andrew NR (2015). Influence of temperature and humidity regimes on the developmental stages of Green Vegetable Bug, *Nezara viridula* (L.) (Hemiptera: Pentatomidae) from inland and coastal populations in Australia. General and Applied Entomology.

[ref-25] Chown SL, Jumbam KR, Sørensen JG, Terblanche JS (2009). Phenotypic variance, plasticity and heritability estimates of critical thermal limits depend on methodological context. Functional Ecology.

[ref-26] Chown SL, Terblanche JS (2007). Physiological diversity in insects: ecological and evolutionary contexts. Advances in Insect Physiology.

[ref-27] Clusella-Trullas S, Blackburn TM, Chown SL (2011). Climatic predictors of temperature performance curve parameters in ectotherms imply complex responses to climate change. American Naturalist.

[ref-28] Coumou D, Rahmstorf S (2012). A decade of weather extremes. Nature Climate Change.

[ref-29] CSIRO-ABM (2012). State of the climate 2012.

[ref-30] Dai A (2011). Drought under global warming: a review. Wiley Interdisciplinary Reviews: Climate Change.

[ref-31] Dell AI, Pawar S, Savage VM (2011). Systematic variation in the temperature dependence of physiological and ecological traits. Proceedings of the National Academy of Sciences of the United States of America.

[ref-32] Del Toro I, Ribbons RR, Pelini SL (2012). The little things that run the world revisited: a review of ant-mediated ecosystem services and disservices (Hymenoptera: Formicidae). Myrmecological News.

[ref-33] Deutsch CA, Tewksbury JJ, Huey RB, Sheldon KS, Ghalambor CK, Haak DC, Martin PR (2008). Impacts of climate warming on terrestrial ectotherms across latitude. Proceedings of the National Academy of Sciences of the United States of America.

[ref-34] Diamond SE, Chick L, Perez A, Strickler SA, Martin RA (2017). Rapid evolution of ant thermal tolerance across an urban-rural temperature cline. Biological Journal of the Linnean Society.

[ref-35] Diamond SE, Sorger DM, Hulcr J, Pelini SL, Toro ID, Hirsch C, Oberg E, Dunn RR (2012). Who likes it hot? A global analysis of the climatic, ecological, and evolutionary determinants of warming tolerance in ants. Global Change Biology.

[ref-36] Doherty MD (1998). The conservation value of regrowth native plant communities: a review.

[ref-37] Dorrough J, Stoi J, McIntyre S (2008). Biodiversity in the paddock: a land manager’s guide.

[ref-38] Duursma R (2017). bootpredictlme4 GitHub repository. https://github.com/remkoduursma.

[ref-39] Ettershank G (1968). The three dimensional gallery structure of the nest of the meat ant Iridomyrmex Purpureus (SM.) (Hymenoptera: Formicidae). Australian Journal of Zoology.

[ref-40] Fattorini S, Salvati L (2014). Tenebrionid beetles as proxy indicators of climate aridity in a Mediterranean area. Ecological Indicators.

[ref-41] Forister ML, McCall AC, Sanders NJ, Fordyce JA, Thorne JH, O’Brien J, Waetjen DP, Shapiro AM (2010). Compounded effects of climate change and habitat alteration shift patterns of butterfly diversity. Proceedings of the National Academy of Sciences of the United States of America.

[ref-42] Frouz J, Jílková V (2008). The effect of ants on soil properties and processes (Hymenoptera: Formicidae). Myrmecological News.

[ref-43] Gaston KJ (2011). Common ecology. BioScience.

[ref-44] Gibb H (2005). The effect of a dominant ant, *Iridomyrmex purpureus*, on resource use by ant assemblages depends on microhabitat and resource type. Austral Ecology.

[ref-45] Gibb H, Hochuli DF (2004). Removal experiment reveals limited effects of a behaviorally dominant species on ant assemblages. Ecology.

[ref-46] Girvetz EH, Zganjar C (2014). Dissecting indices of aridity for assessing the impacts of global climate change. Climatic Change.

[ref-47] Greaves T (1971). The distribution of the three forms of the meat ant *Iridomyrmex purpureus* (Hymenoptera: Formicidae) in Australia. Australian Journal of Entomology.

[ref-48] Greaves T, Hughes RD (1974). The population biology of the meat ant. Australian Journal of Entomology.

[ref-49] Greenslade PJM (1975). Dispersion and history of a population of the meat ant *Iridomyrmex purpureus* (Hymenoptera: Formicidae). Australian Journal of Zoology.

[ref-50] Greenslade PJM (1976). The meat ant *Iridomyrmex purpureus* (Hymenoptera: Formicidae) as a dominant member of ant communities. Australian Journal of Entomology.

[ref-51] Grigaltchik VS, Ward AJW, Seebacher F (2012). Thermal acclimation of interactions: differential responses to temperature change alter predator-prey relationship. Proceedings of the Royal Society B: Biological Sciences.

[ref-52] Harris RMB, Beaumont LJ, Vance TR, Tozer CR, Remenyi TA, Perkins-Kirkpatrick SE, Mitchell PJ, Nicotra AB, McGregor S, Andrew NR, Letnic M, Kearney MR, Wernberg T, Hutley LB, Chambers L-E, Fletcher MS, Keatley MR, Woodward CA, Williamson G, Duke NC, Bowman DMJS (2018). Biological responses to the press and pulse of climate trends and extreme events. Nature Climate Change.

[ref-53] Hemmings Z, Andrew NR (2017). Effects of microclimate and species identity on body temperature, and thermal tolerance, of ants (Hymenoptera: Formicidae). Austral Entomology.

[ref-54] Hoffmann AA, Chown SL, Clusella-Trullas S (2013). Upper thermal limits in terrestrial ectotherms: How constrained are they?. Functional Ecology.

[ref-55] Hoffmann AA, Sgro CM (2011). Climate change and evolutionary adaptation. Nature.

[ref-56] Hölldobler B, Wilson EO (1990). The ants.

[ref-57] Huang S-P, Talal S, Ayali A, Gefen E (2015). The effect of discontinuous gas exchange on respiratory water loss in grasshoppers (Orthoptera: Acrididae) varies across an aridity gradient. Journal of Experimental Biology.

[ref-58] Huey RB, Kearney MR, Krockenberger A, Holtum JAM, Jess M, Williams SE (2012). Predicting organismal vulnerability to climate warming: roles of behaviour, physiology and adaptation. Philosophical Transactions of the Royal Society B: Biological Sciences.

[ref-59] Inger R, Gregory R, Duffy JP, Stott I, Voříšek P, Gaston KJ (2014). Common European birds are declining rapidly while less abundant species’ numbers are rising. Ecology Letters.

[ref-60] Kearney RM (2013). Activity restriction and the mechanistic basis for extinctions under climate warming. Ecology Letters.

[ref-61] Kearney MR, Deutscher J, Kong JD, Hoffmann AA (2018). Summer egg diapause in a matchstick grasshopper synchronizes the life cycle and buffers thermal extremes. Integrative Zoology.

[ref-62] Keith DA (2004). Ocean shores to desert dunes: the native vegetation of New South Wales and the ACT.

[ref-63] Kingsolver JG, Diamond SE, Buckley LB (2013). Heat stress and the fitness consequences of climate change for terrestrial ectotherms. Functional Ecology.

[ref-64] Kingsolver JG, Umbanhowar J (2018). The analysis and interpretation of critical temperatures. Journal of Experimental Biology.

[ref-65] Laurance WF, Williamson GB (2001). Positive feedbacks among forest fragmentation, drought, and climate change in the Amazon. Conservation Biology.

[ref-66] Lighton JR, Turner RJ (2004). Thermolimit respirometry: an objective assessment of critical thermal maxima in two sympatric desert harvester ants, *Pogonomyrmex rugosus* and *P. californicus*. Journal of Experimental Biology.

[ref-67] Lister BC, Garcia A (2018). Climate-driven declines in arthropod abundance restructure a rainforest food web. Proceedings of the National Academy of Sciences of the United States of America.

[ref-68] Lüdecke D (2017). https://CRAN.R-project.org/package=sjPlot.

[ref-69] Mawdsley JR, O’Malley R, Ojima DS (2009). A review of climate-change adaptation strategies for wildlife management and biodiversity conservation. Conservation Biology.

[ref-70] Mobbs CJ, Tedder G, Wade AM, Williams R (1978). A note on food and foraging in relation to temperature in the meat ant *Iridomyrmex purpureus* form *viridiaeneus*. Australian Journal of Entomology.

[ref-71] Monaenkova D, Gravish N, Rodriguez G, Kutner R, Goodisman MAD, Goldman DI (2015). Behavioral and mechanical determinants of collective subsurface nest excavation. Journal of Experimental Biology.

[ref-72] Mooney H, Larigauderie A, Cesario M, Elmquist T, Hoegh-Guldberg O, Lavorel S, Mace GM, Palmer M, Scholes R, Yahara T (2009). Biodiversity, climate change, and ecosystem services. Current Opinion in Environmental Sustainability.

[ref-73] Nguyen C, Bahar MH, Baker G, Andrew NR (2014). Thermal tolerance limits of diamondback moth in ramping and plunging assays. PLOS ONE.

[ref-74] Nyamukondiwa C, Terblanche JS (2009). Thermal tolerance in adult Mediterranean and Natal fruit flies (*Ceratitis capitata* and *Ceratitis rosa*): Effects of age, gender and feeding status. Journal of Thermal Biology.

[ref-75] Oberst S, Lai JCS, Evans TA (2016). Termites utilise clay to build structural supports and so increase foraging resources. Scientific Reports.

[ref-76] O’Geen AT (2013). Soil water dynamics. Nature Education Knowledge.

[ref-77] Oliver I, Dorrough J, Doherty H, Andrew NR (2016). Additive and synergistic effects of land cover, land use and climate on insect biodiversity. Landscape Ecology.

[ref-78] Oliver TH, Morecroft MD (2014). Interactions between climate change and land use change on biodiversity: attribution problems, risks, and opportunities. Wiley Interdisciplinary Reviews: Climate Change.

[ref-79] Pérez-Sánchez AJ, Lattke JE, Viloria AL (2013). Patterns of Ant (Hymenoptera: Formicidae) Richness and Relative Abundance along an Aridity Gradient in Western Venezuela. Neotropical Entomology.

[ref-80] Picanço A, Rigal F, Matthews TJ, Cardoso P, Borges PAV (2017). Impact of land-use change on flower-visiting insect communities on an oceanic island. Insect Conservation and Diversity.

[ref-81] Pörtner HO, Farrell AP (2008). Physiology and climate change. Science.

[ref-82] Punzo F (1991). The effects of temperature and moisture on survival capacity, cuticular permeability, hemolymph osmoregulation and metabolism in the scorpion, *Centruroides hentzi* (banks) (scorpiones, buthidae). Comparative Biochemistry and Physiology Part A: Physiology.

[ref-83] Punzo F, Mutchmor JA (1980). Effects of temperature, relative humidity and period of exposure on the survival capacity of *Tenebrio molitor* (Coleoptera: Tenebrionidae). Journal of the Kansas Entomological Society.

[ref-84] R Core Team (2017). R: a language and environment for statistical computing.

[ref-85] Sala OE, Stuart Chapin F, Armesto JJ, Berlow E, Bloomfield J, Dirzo R, Huber-Sanwald E, Huenneke LF, Jackson RB, Kinzig A, Leemans R, Lodge DM, Mooney HA, Oesterheld M, Poff NL, Sykes MT, Walker BH, Walker M, Wall DH (2000). Global biodiversity scenarios for the year 2100. Science.

[ref-86] Saxton KE, Rawls WJ, Romberger JS, Papendick RI (1986). Estimating generalized soil-water characteristics from texture. Soil Science Society of America Journal.

[ref-87] Scherer C, Jeltsch F, Grimm V, Blaum N (2016). Merging trait-based and individual-based modelling: An animal functional type approach to explore the responses of birds to climatic and land use changes in semi-arid African savannas. Ecological Modelling.

[ref-88] Sinclair BJ, Marshall KE, Sewell MA, Levesque DL, Willett CS, Slotsbo S, Dong Y, Harley CDG, Marshall DJ, Helmuth BS, Huey RB (2016). Can we predict ectotherm responses to climate change using thermal performance curves and body temperatures?. Ecology Letters.

[ref-109] Smith F (1858). Catalogue of hymenopterous insects in the collection of the British Museum. Part VI. Formicidae.

[ref-89] Smith MD (2011a). An ecological perspective on extreme climatic events: a synthetic definition and framework to guide future research. Journal of Ecology.

[ref-90] Smith MD (2011b). The ecological role of climate extremes: current understanding and future prospects. Journal of Ecology.

[ref-91] Sørensen JG, Dahlgaard J, Loeschcke V (2001). Genetic variation in thermal tolerance among natural populations of *Drosophila buzzatii*: down regulation of Hsp70 expression and variation in heat stress resistance traits. Functional Ecology.

[ref-92] Stephenson NL (1998). Actual evapotranspiration and deficit: Biologically meaningful correlates of vegetation distribution across spatial scales. Journal of Biogeography.

[ref-93] Stuble KL, Patterson CM, Rodriguez-Cabal MA, Ribbons RR, Dunn RR, Sanders NJ (2014). Ant-mediated seed dispersal in a warmed world. PeerJ.

[ref-94] Suhling F, Martens A, Marais E (2009). How to enter a desert—patterns of Odonata colonisation of arid Namibia. International Journal of Odonatology.

[ref-95] Sunday JM, Bates AE, Kearney MR, Colwell RK, Dulvy NK, Longino JT, Huey RB (2014). Thermal-safety margins and the necessity of thermoregulatory behavior across latitude and elevation. Proceedings of the National Academy of Sciences of the United States of America.

[ref-96] Terblanche JS, Deere JA, Clusella-Trullas S, Janion C, Chown SL (2007). Critical thermal limits depend on methodological context. Proceedings of the Royal Society B: Biological Sciences.

[ref-97] Traill LW, Lim MLM, Sodhi NS, Bradshaw CJA (2010). Mechanisms driving change: altered species interactions and ecosystem function through global warming. Journal of Animal Ecology.

[ref-98] Travis JMJ (2003). Climate change and habitat destruction: a deadly anthropogenic cocktail. Proceedings of the Royal Society of London Series B: Biological Sciences.

[ref-99] Tuck SL, Winqvist C, Mota F, Ahnström J, Turnbull LA, Bengtsson J (2014). Land-use intensity and the effects of organic farming on biodiversity: a hierarchical meta-analysis. Journal of Applied Ecology.

[ref-100] VanDerWal J, Murphy HT, Kutt AS, Perkins GC, Bateman BL, Perry JJ, Reside AE (2013). Focus on poleward shifts in species’ distribution underestimates the fingerprint of climate change. Nature Climate Change.

[ref-101] Vasseur DA, DeLong JP, Gilbert B, Greig HS, Harley CDG, McCann KS, Savage V, Tunney TD, O’Connor MI (2014). Increased temperature variation poses a greater risk to species than climate warming. Proceedings of the Royal Society B: Biological Sciences.

[ref-102] Wiens JJ, Kozak KH, Silva N (2013). Diversity and niche evolution along aridity gradients in north American lizards (phrynosomatidae). Evolution.

[ref-103] Williams CM, Henry HAL, Sinclair BJ (2014). Cold truths: how winter drives responses of terrestrial organisms to climate change. Biological Reviews.

[ref-104] Xu T, Hutchinson MN (2011). ANUCLIM version 6, users guide.

[ref-105] Yates M, Andrew NR (2011). Comparison of ant community composition across different land-use types: assessing morphological traits with more common methods. Australian Journal of Entomology.

[ref-106] Yates ML, Andrew NR, Binns M, Gibb H (2014). Morphological traits: predictable responses to macrohabitats across a 300 km scale. PeerJ.

[ref-107] Yates ML, Gibb H, Andrew NR (2011). Habitat characteristics may override climatic influences on ant assemblage composition: a study using a 300-km climatic gradient. Australian Journal of Zoology.

[ref-108] Yin Y, Ma D, Wu S (2018). Climate change risk to forests in China associated with warming. Scientific Reports.

